# Establishment of a novel weight reduction model after laparoscopic sleeve gastrectomy based on abdominal fat area

**DOI:** 10.3389/fsurg.2024.1390045

**Published:** 2024-05-17

**Authors:** Tianyi Feng, Sanyuan Hu, Changrong Song, Mingwei Zhong

**Affiliations:** ^1^ Department of General Surgery, Shandong Provincial Qianfoshan Hospital, Cheeloo College of Medicine, Shandong University, Jinan, Shandong Province, China; ^2^ Department of General Surgery, Qilu Hospital, Cheeloo College of Medicine, Shandong University, Jinan, Shandong Province, China; ^3^ Department of General Surgery, The First Affiliated Hospital of Shandong First Medical University, Shandong Provincial Qianfoshan Hospital, Jinan, Shandong Province, China

**Keywords:** total abdominal fat area, visceral fat area (VFA), bariatric surgery, sleeve gastrectomy, obesity, weight loss

## Abstract

In light of ongoing research elucidating the intricacies of obesity and metabolic syndrome, the role of abdominal fat (especially visceral fat) has been particularly prominent. Studies have revealed that visceral adipose tissue can accelerate the development of metabolic syndrome by releasing various bioactive compounds and hormones, such as lipocalin, leptin and interleukin. A retrospective analysis was performed on the clinical data of 167 patients with obesity. Among them, 105 patients who satisfied predefined inclusion and exclusion criteria were included. The parameters evaluated included total abdominal fat area (TAFA), laboratory indicators and anthropometric measurements. Weight reduction was quantified through percent total weight loss (%TWL) and percent excess weight loss (%EWL) postoperatively. Binary logistic regression analysis and receiver operating characteristic (ROC) curve analysis were employed to identify predictors of weight loss. Binary logistic regression analysis emphasized that total abdominal fat area was an independent predictor of %EWL ≥75% (*p* < 0.001). Total abdominal fat area (*p* = 0.033) and BMI (*p* = 0.003) were independent predictors of %TWL ≥30%. In our cohort, %TWL ≥30% at 1 year after surgery was closely related to the abdominal fat area and BMI. Based on these results, we formulated a novel model based on these factors, exhibiting superior predictive value for excellent weight loss.

## Introduction

Obesity is a chronic disease caused by excessive accumulation of fat in the body due to the interaction of genetic, environmental and endocrine factors. Obesity is also an important cause of many metabolic diseases, such as type 2 diabetes, fatty liver and hypertension. It has now become a major global public health problem (1, 2).

As an effective means to combat obesity and metabolic syndrome (3, 4), bariatric surgery mainly includes Roux-en-Y gastric bypass (RYGB), and sleeve gastrectomy (SG). In recent years, due to its low complication rate and the revelation of metabolic mechanism, SG has become the most common surgical procedure in North America, the Asia-Pacific region and even the world (5–7). The outcomes of weight reduction after bariatric surgery exhibit considerable variance because of different preoperative parameters in different patients, such as C-peptide, body mass index (BMI), insulin levels, and fasting blood glucose (FBG) levels.

Recent studies have shown that the regional distribution of adipose tissue is critical in the evolution of obesity (8). Abdominal obesity is characterized by an excessive accumulation of visceral fat. Patients with abdominal obesity tend to have more severe abnormalities in glucose-lipid metabolism. Numerous studies have established a robust correlation between visceral adiposity and insulin resistance, vascular endothelial dysfunction, and related comorbidities (9–11). Above all, studies reveal that abdominal adipose tissue plays an indispensable role in development of obesity. However, the association between abdominal fat area and effect of weight loss postoperatively is still unclear. Thus, this study investigated the association between weight loss after LSG and abdominal fat area or other preoperative factors.

## Materials and methods

### Subject

**Figure 1:**
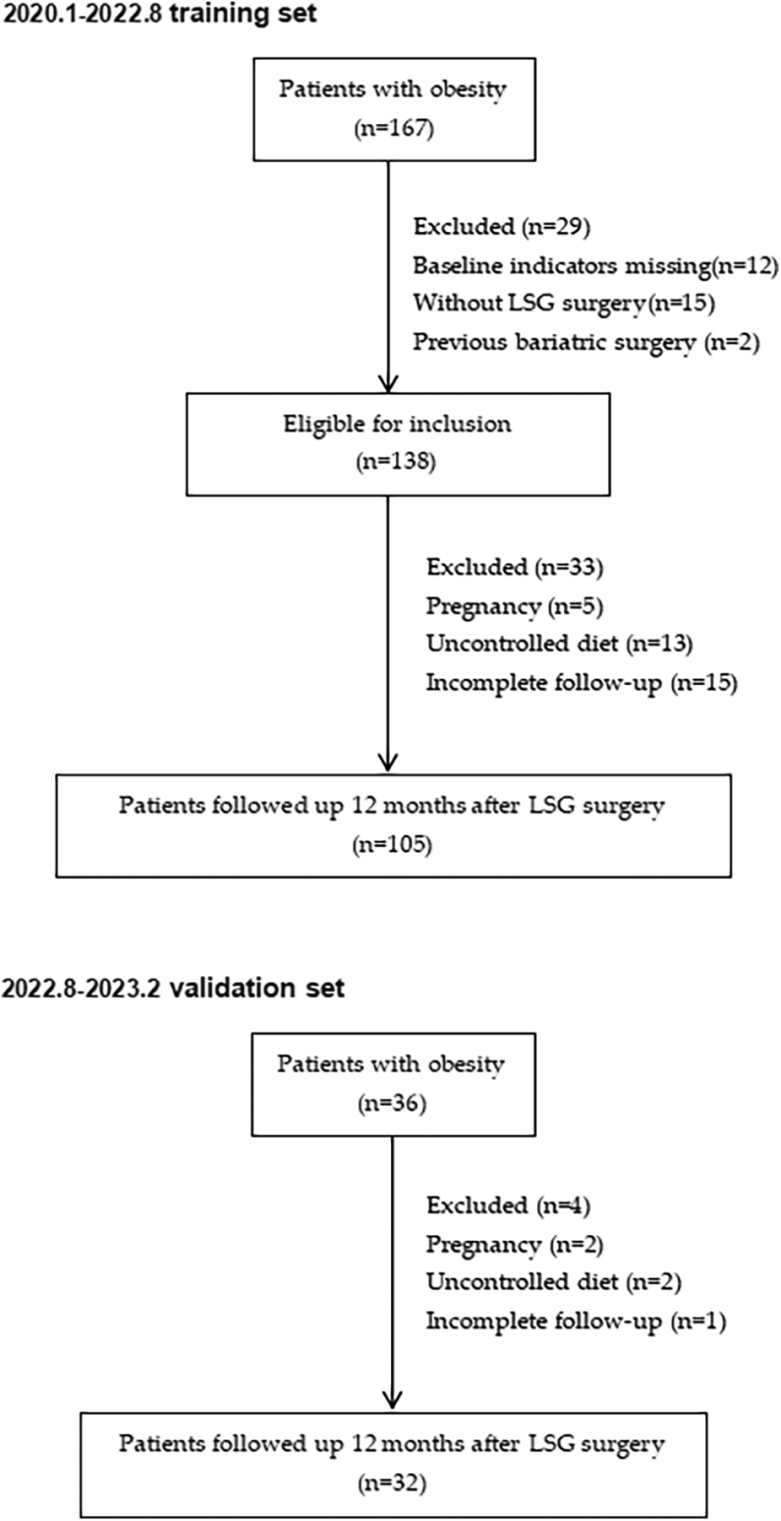
Follow-up study flow chart on patients who underwent LSG.

A retrospective study was conducted encompassing 167 patients with obesity from January 2020 to August 2022 at our medical center (Figure 1). Then, the clinical data of 32 patients who underwent sleeve gastrectomy between August 2022 and February 2023 were collected as the validation cohort to verify the accuracy of the model. In adherence to the guidelines for bariatric surgery (12), preoperative assessments encompass ultrasonography, computed tomography (CT), sleep apnea surveillance, bone mineral density evaluations, and other diagnostic modalities. These meticulous examinations are systematically conducted as integral components of the pre-surgical regimen, aiming to delineate surgical indications while systematically eliminating potential contraindications. Inclusion criteria encompassed (1) BMI ≥ 32.5 kg/m^2^ in the absence of comorbidities, or BMI ≥ 27.5 kg/m^2^ with diabetes mellitus or other metabolic syndromes (13, 14) (2) the presence of complete preoperative imaging data (3) the patients who underwent laparoscopic sleeve gastrectomy(LSG) with complete postoperative follow-up data. Key exclusion criteria included (1) secondary obesity, such as hypothyroidism, and acromegaly. (2) Patients are unable to tolerate surgery or anesthesia due to poor cardiovascular and other system functions, or have uncontrolled mental illness (3) Patients with poor postoperative nutritional compliance and who do not follow the dietary guidance provided by doctors and nutritionists.

### Indicators

Anthropometric parameters as well as serologic indicators were comprehensively included in this study, where anthropometric parameters mainly included age, gender, BMI and serologic indicators mainly encompassed thyroid function, liver function, and glycosylated hemoglobin. Comorbidities mainly included hyperlipidemia, type 2 diabetes, and hypertension. Hypertension was defined as blood pressure ≥140/90 mmHg (15). The primary outcome variable, on the other hand, was the percent excess weight loss (%EWL) and percent total weight loss (%TWL) 1 year post-surgery. Percent total weight loss (%TWL) was calculated using the formula: Weight loss/(preoperative weight) × 100%. %EWL is usually defined as follows: %EWL = (weight loss/baseline excess weight) × 100%, and baseline excess weight = baseline weight—ideal weight. The ideal weight is based on the person's weight at a BMI of 25 kg/m^2^ (16–20). Excellent weight loss was defined as %TWL ≥30% or %EWL ≥75% (17, 21).

### Measurement of abdominal fat area

**Figure 2:**
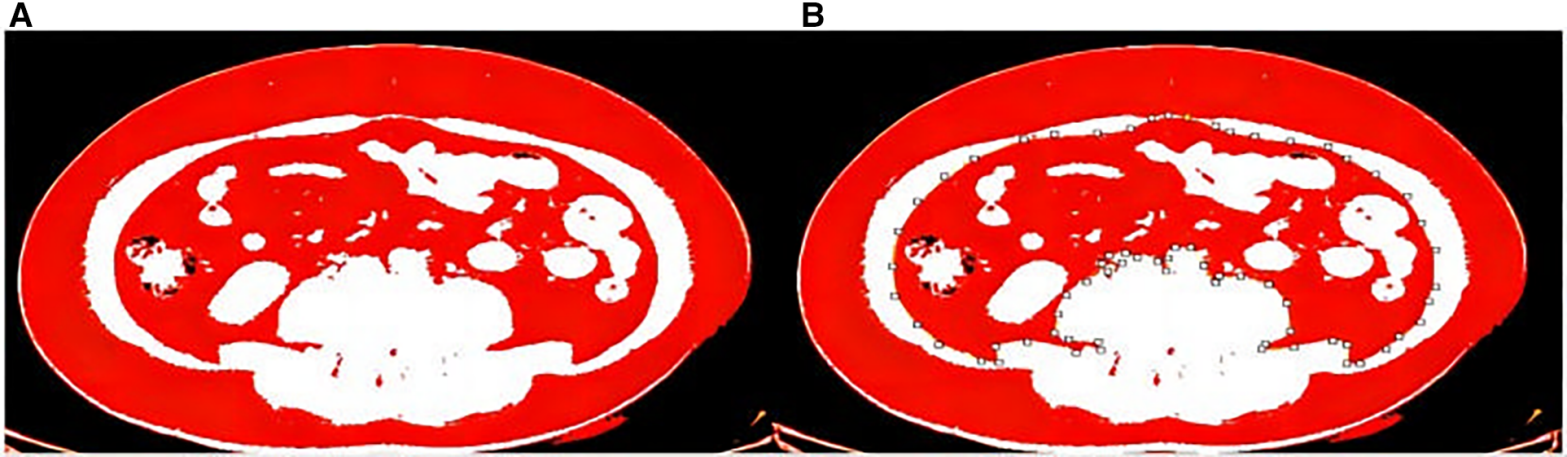
(**A**) Total abdominal fat area. (**B**) Visceral fat area.

In this study, Image J software was used for measurement. The Hounsfield threshold of adipose tissue in CT is −190 to −30 HU (22, 23), according to which adipose tissue can be successfully visualized by adjusting the specific window position and width of CT. According to related studies (24), the abdominal fat area in the third lumbar vertebrae plane is more representative of the abdominal fat content. Therefore, this study measured the fat area in the L3 plane for analysis and processing, and measured the total abdominal fat area, visceral fat area, subcutaneous fat area, and the ratio of visceral fat and subcutaneous fat, respectively (Figures 2A,B).

### Statistical methods

We performed correlation analyses between preoperative variables and %EWL/%TWL, with Pearson's correlation analysis for variables conforming to a normal distribution and Spearman's correlation analysis for non-normally distributed variables, whereby independent factors were identified. Backward linear stepwise regression was used to identify independent predictors associated with weight loss 1 year after bariatric surgery. Binary logistic regression was used for predicting dependent variables. All statistical analyses were performed using SPSS26.0. ROC curve was performed for assessing model discrimation, and AUC, sensitivity, specificity, and Jordon's index were calculated. Bootstrap method was used to test the calibration of the model, while the Hosmer-Lemeshow test was used to determine the goodness of fit of the model. The clinical utility of the model was evaluated using R4.2.0 software to plot decision curve analysis (DCA).

## Results

### Association between weight reduction with fat area

**Figure 3:**
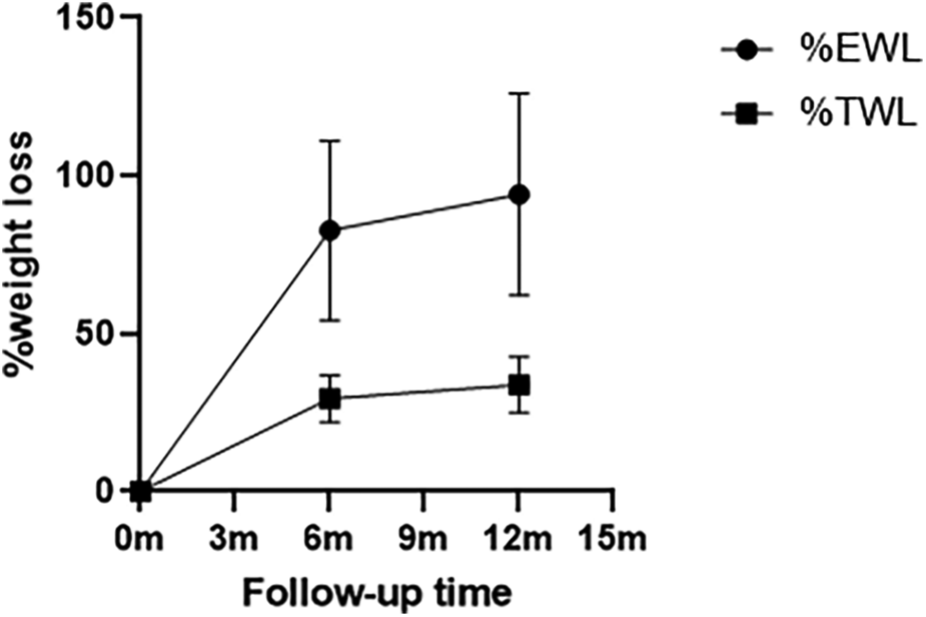
Weight loss plot over time. Values are shown as the mean values of %EWL by the circle dots and %TWL by the square dots, and standard deviation of both by vertical lines.

One hundred and five patients with obesity completed the 6-month follow-up, achieving a mean loss of 82.72% of excess weight loss and 29.50% of their total weight loss. The maximum weight loss was observed at 1 year post-surgery, with 94.09%EWL and 33.83%TWL (Figure 3). As shown in Table 1, parameters associated with %TWL and %EWL included BMI, TAFA, creatinine, cystatin C during the 6 months follow-up period. And parameters correlated with %TWL included BMI, TAFA, subcutaneous fat area and creatinine 1 year after surgery. Research has also found that such parameters (BMI, TAFA, VFA, SFA, V/S ratio, cystatin C, β2 microglobulin, uric acid, and homocysteine) were negatively correlated with %EWL 1 year postoperatively, while preoperative SOD levels were positively correlated with it. Therefore, based on the above results, a multiple linear regression analysis further highlighted that TAFA, V/S ratio, and superoxide dismutase were independently associated with %EWL at the 12-month postoperative follow-up (Table 2).

**Table 1:** Associations of weight loss effect parameters with pre-operative parameters.

Variable	6 months				12 months			
	%EWL		%TWL		%EWL		%TWL	
	*r*	*p*	*r*	*p*	*r*	*p*	*r*	*p*
Age	0.134	0.173	0.053	0.592	−0.038	0.704	−0.192	0.05
BMI	−0.741	<0.01**	0.264	<0.01**	−0.651	<0.01**	0.385	<0.01**
TAFA	−0.621	<0.01**	0.199	0.042*	−0.587	<0.01**	0.257	<0.01**
VFA	−0.571	<0.01**	0.154	0.117	−0.581	<0.01**	0.168	0.086
SFA	−0.546	<0.01**	0.188	0.055	−0.496	<0.01**	0.261	<0.01**
VFA/SFA	−0.173	0.078	0.035	0.727	−0.215	0.028*	−0.007	0.943
SOD	0.168	0.087	−0.087	0.377	0.316	<0.01**	0.133	0.175
Glycocholic acid	−0.035	0.732	0.202	0.043*	−0.087	0.389	0.104	0.302
Creatinine	−0.241	0.013*	0.223	0.022*	−0.189	0.053	0.250	0.01*
Cystatin C	−0.379	<0.01**	0.195	0.046*	−0.375	<0.01**	0.174	0.076
β2-microglobulin	−0.477	<0.01**	0.028	0.777	−0.415	<0.01**	0.092	0.351
Uric acid	−0.323	<0.01**	0.036	0.716	−0.322	<0.01**	0.074	0.456
Triglyceride	−0.118	0.23	−0.05	0.61	−0.132	0.18	−0.061	0.536
Homocysteine	−0.340	<0.01**	0.107	0.279	−0.299	<0.01**	0.139	0.158
Testosterone	−0.155	0.115	0.250	<0.01**	−0.158	0.108	0.251	<0.01**
Estradiol	0.039	0.689	0.141	0.151	−0.002	0.985	0.014	0.89

%EWL, percent extra weight loss; %TWL, percent total weight loss; TAFA, total abdominal fat area; VFA, visceral fat area; SFA, subcutaneous fat area; SOD, superoxide dismutase.

* *p* < 0.05; **p < 0.01.

**Table 2:** Multiple linear regression of %EWL (12 months) with pre-operative parameters.

%EWL	12 months		
	Standardized coefficients *β*	*t*	*p*
TAFA	−1.055	−3.496	0.001**
VFA	0.734	1.967	0.052
VFA/SFA	−0.574	−2.607	0.011*
SOD	0.237	2.961	0.004**

*p < 0.05; **p < 0.01; ***p < 0.001.

### Abdominal fat area was an independent predictor of excellent weight reduction

Excellent weight loss was defined as %TWL ≥30% or %EWL ≥75% (17, 21). Firstly, the patients who underwent LSG were divided into 2 groups according to %EWL ≥75% (Table 3). Upon analyzing baseline metrics, it was shown that BMI, TAFA, VFA, SFA, SOD, cystatin C, β2-microglobulin, uric acid, homocysteine, apolipoprotein A1, and gender emerged as independent predictors of excellent weight loss and that metrics were included in the logistic regression analysis, only TAFA, SOD and apolipoprotein A1 were included, while SOD and apolipoprotein A1 were not statistically significant (*p* > 0.05)(Table 4). Therefore, it was determined that a lower abdominal fat area independently predicted excellent weight loss outcomes 1 year postoperatively in our cohort [OR: 0.996 (95% confidence interval: 0.993–0.998), *p* = 0.001].

**Table 3:** Characteristics between patients grouped by %EWL ≥75%.

Variable	Classification				
	Total	%EWL <75%	%EWL >75%	*t*/Z/*χ*²	*p*
Age (year)	31.0 (24.5, 36.0)	33.0 (26.0, 38.0)	31 (24, 36)	−0.730	0.465
BMI (kg/m^2^)	40.8 (35.3, 46.9)	47.6 (40.8, 50.0)	38.9 (34.8, 43.9)	−3.966	<0.001***
TAFA (cm^2^)	628.5 (502.1, 815.0)	834.0 (560.8, 959.5)	585.2 (473.7, 733.8)	−3.798	<0.001***
VFA (cm^2^)	222.7 (141.6, 275.4)	262.3 (222.3, 309.2)	200.7 (133.1, 259.1)	−3.461	0.001**
SFA (cm^2^)	438.2 ± 135.9	513.2 ± 149.6	412.2 ± 121.3	3.507	0.001**
VFA/SFA	0.5 (0.4, 0.7)	0.5 (0.4, 0.7)	0.5 (0.4, 0.6)	−0.843	0.399
SOD (U/ml)	167.4 ± 17.0	160.4 ± 14.4	169.8 ± 17.3	−2.540	0.013*
Creatinine (umol/L)	62.0 (52.5, 70.5)	62.0 (54.0, 72.0)	60.5 (52.0, 70.0)	−0.873	0.383
Cystatin C (mg/L)	0.8 (0.7, 0.9)	0.8 (0.7, 1.0)	0.8 (0.7, 0.9)	−2.171	0.030*
β2-microglobulin (mg/L)	1.7 (1.5, 1.9)	1.8 (1.6, 2.1)	1.7 (1.5, 1.9)	−2.409	0.016*
Uric Acid (umol/L)	409.0 (350.0, 491.0)	450.0 (369.0, 547.0)	402.0 (342.0, 476.0)	−2.086	0.037*
Apolipoprotein A1(mg/L)	871.5 ± 110.7	827.4 ± 110.4	886.8 ± 107.2	−2.426	0.019*
Testosterone (ng/ml)	0.5 (0.4, 1.8)	1.0 (0.4, 2.0)	0.5 (0.4, 1.2)	−0.722	0.470
Estradiol (ng/L)	47.5 (34.6, 69.9)	49.3 (34.3, 63.2)	47.0 (34.7, 70.5)	−0.099	0.921
Gender (men/women)	35/70	14/13	21/57	5.609	0.018*
Metabolic syndrome (%)	52.4	63.0	48.7	1.632	0.201
Hypertension (%)	36.2	44.4	33.3	1.072	0.300
T2DM (%)	45.7	48.1	44.9	0.087	0.768

*p < 0.05; **p < 0.01; ***p < 0.001.

**Table 4:** Regression of binary logistic of more than 75% excess weight loss (12 months) with pre-operative parameters.

%EWL	12 months	
	OR (95% CI)	*p*
TAFA	0.996 (0.993, 0.998)	0.001**
SOD	1.029 (0.999, 1.060)	0.058
Apolipoprotein A1	1.005 (0.999, 1.010)	0.079

*p < 0.05; **p < 0.01; ***p < 0.001.

Then, patients were stratified into two distinct groups predicated on the criterion of achieving %TWL ≥30% (Table 5). BMI, TAFA, SFA, HDL cholesterol and testosterone were independent predictors of excellent weight loss. Subsequently, a rigorous binary logistic regression analysis was conducted. Remarkably, only BMI and TAFA retained their stature as independent predictors of the attainment of a %TWL ≥30% 1 year postoperatively in our cohort (Table 6). Of particular note, the statistical analysis revealed that TAFA exhibited a formidable and robust predictive efficacy in relation to both %TWL and %EWL.

**Table 5:** Characteristics between patients grouped by %TWL ≥30%.

Variable	Classification				
	Total	%TWL <30%	%TWL >30%	*t*/*Z*/*χ*²	*p*
Age (year)	31.5 ± 8.6	34.7 ± 10.7	30.0 ± 7.1	2.287	0.027*
BMI (kg/m^2^)	40.8 (35.3, 46.9)	35.4 (33.7, 42.6)	42.8 (37.9, 47.4)	−3.551	<0.001***
TAFA (cm^2^)	628.5 (502.1, 815.0)	548.5 (471.0, 735.2)	681.1 (527.3, 840.6)	−2.000	0.046*
VFA (cm^2^)	222.7 (141.6, 275.4)	218.5 (147.1, 254.9)	226.4 (140.8, 289.2)	−1.000	0.317
SFA (cm^2^)	434.7 (333.6, 543.4)	357.6 (310.9, 452.6)	471.9 (356.8, 544.8)	−2.349	0.019*
VFA/SFA	0.5 (0.4, 0.7)	0.5 (0.4, 0.7)	0.5 (0.4, 0.6)	−0.582	0.561
SOD (U/ml)	167.39 ± 17.0	164.7 ± 16.6	168.7 ± 17.2	−1.121	0.265
Creatinine (umol/L)	62.0 (52.5, 70.5)	59.5 (47.0, 67.8)	62.0 (54.0, 72.0)	−1.901	0.057
Cystatin C(mg/L)	0.8 (0.7, 0.9)	0.7 (0.6, 0.9)	0.8 (0.7, 0.9)	−1.422	0.155
β2-microglobulin(mg/L)	1.7 (1.5, 1.9)	1.6 (1.5, 1.9)	1.7 (1.6, 2.0)	−1.099	0.272
Uric acid (umol/L)	425.1 ± 110.2	413.2 ± 111.7	430.8 ± 109.8	−0.763	0.447
Testosterone (ng/ml)	0.5 (0.4, 1.8)	0.4 (0.3, 0.7)	0.6 (0.4, 2.0)	−2.431	0.015*
Estradiol (ng/L)	47.5 (34.6, 69.9)	48.6 (30.9, 80.4)	46.6 (35.1, 69.5)	−0.243	0.808
Gender (men/women)	35/70	7/27	28/43	3.675	0.055
Metabolic syndrome (%)	52.4	52.9	52.1	0.006	0.937
Hypertension (%)	36.2	44.1	32.4	1.368	0.242
T2DM (%)	45.7	47.1	45.1	0.037	0.848

*p < 0.05; **p < 0.01; ***p < 0.001.

**Table 6:** Regression of binary logistic of more than 30% total weight loss (12 months) with pre-operative parameters.

%TWL	12 months	
	OR (95% CI)	*p*
Age	0.948 (0.897, 1.001)	0.055
BMI	1.243 (1.078, 1.433)	0.003**
TAFA	0.994 (0.989, 1.000)	0.033*
Testosterone	1.593 (0.915, 2.772)	0.099

*p < 0.05; **p < 0.01; ***p < 0.001.

### The regression equation to predict weight loss effect (%TWL ≥ 30%)

**Figure 4:**
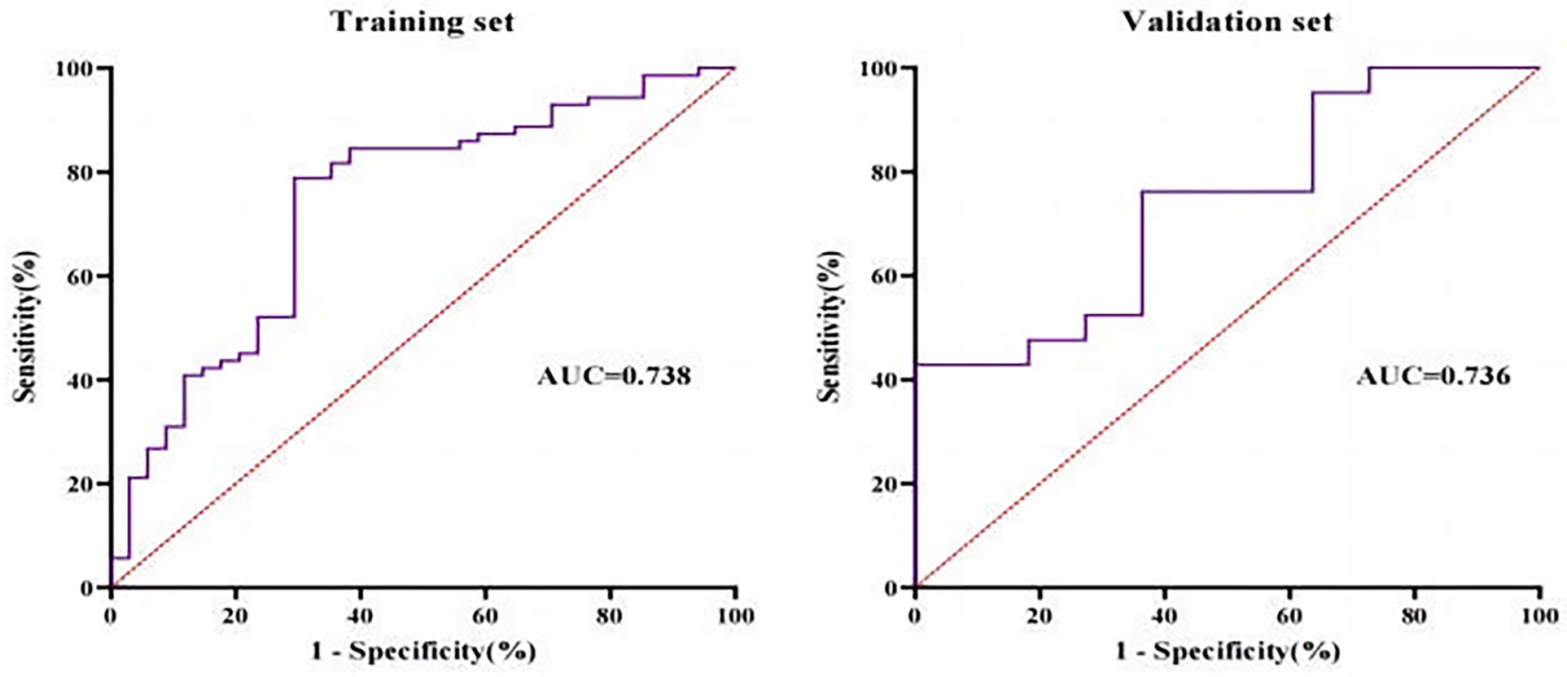
Receiver-operating characteristic (ROC) for training set and validation set.

Based on the above results of logistic regression analysis, the regression equation for the model was derived as logit (*P*) = 0.212 × BMI-0.004 × TAFA-5.419. In the training set, for predicting %TWL ≥30%, the prediction model exhibited an area under the curve (AUC) of 0.738 (*p* < 0.001), with a sensitivity of 78.9%, specificity of 70.6%, and Yoden index of 0.495. As for validation set, the model had an AUC of 0.736 for predicting %TWL ≥30% (*p* = 0.031), sensitivity of 42.9%, specificity of 100.0% and a Yoden index of 0.429 (Figure 4).

### Assessment of calibration and clinical benefit of prediction models

**Figure 5:**
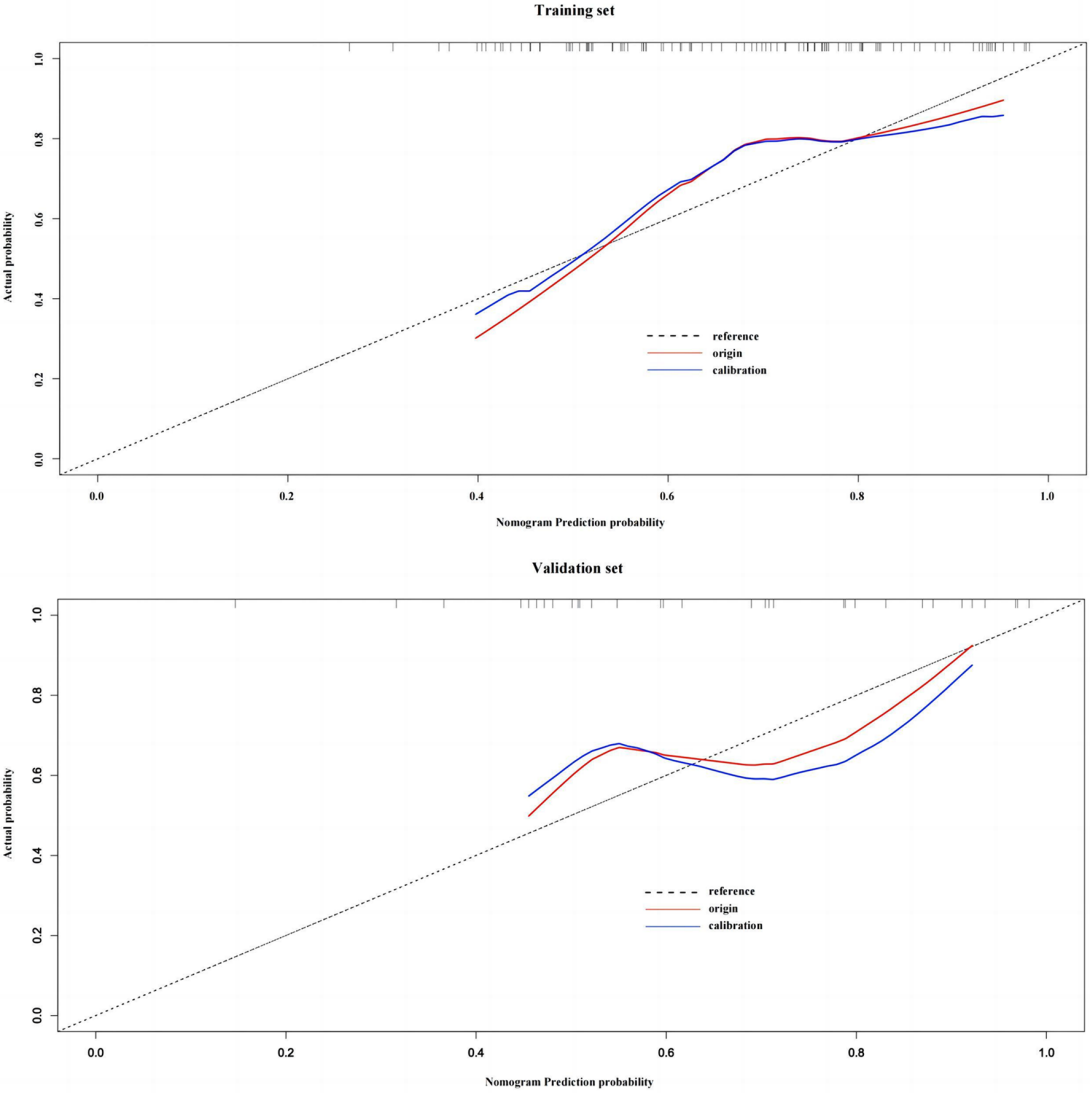
Model calibration plots for training set and validation set.

The Hosmer-Lemeshow test found that the difference was not statistically significant when comparing the actual %TWL ≥30% occurrence probability and the predicted probability in the training set (*χ*^2 ^= 13.062, *df *= 8, *p *= 0.110). In the validation set, the difference was also not statistically significant (*χ*^2 ^= 15.184, *df *= 8, *p *= 0.056). After using 1,000 bootstrap models, model calibration plots show good agreement between predictive model and actual clinical observations and had a good calibration degree (See Figure 5).

**Figure 6:**
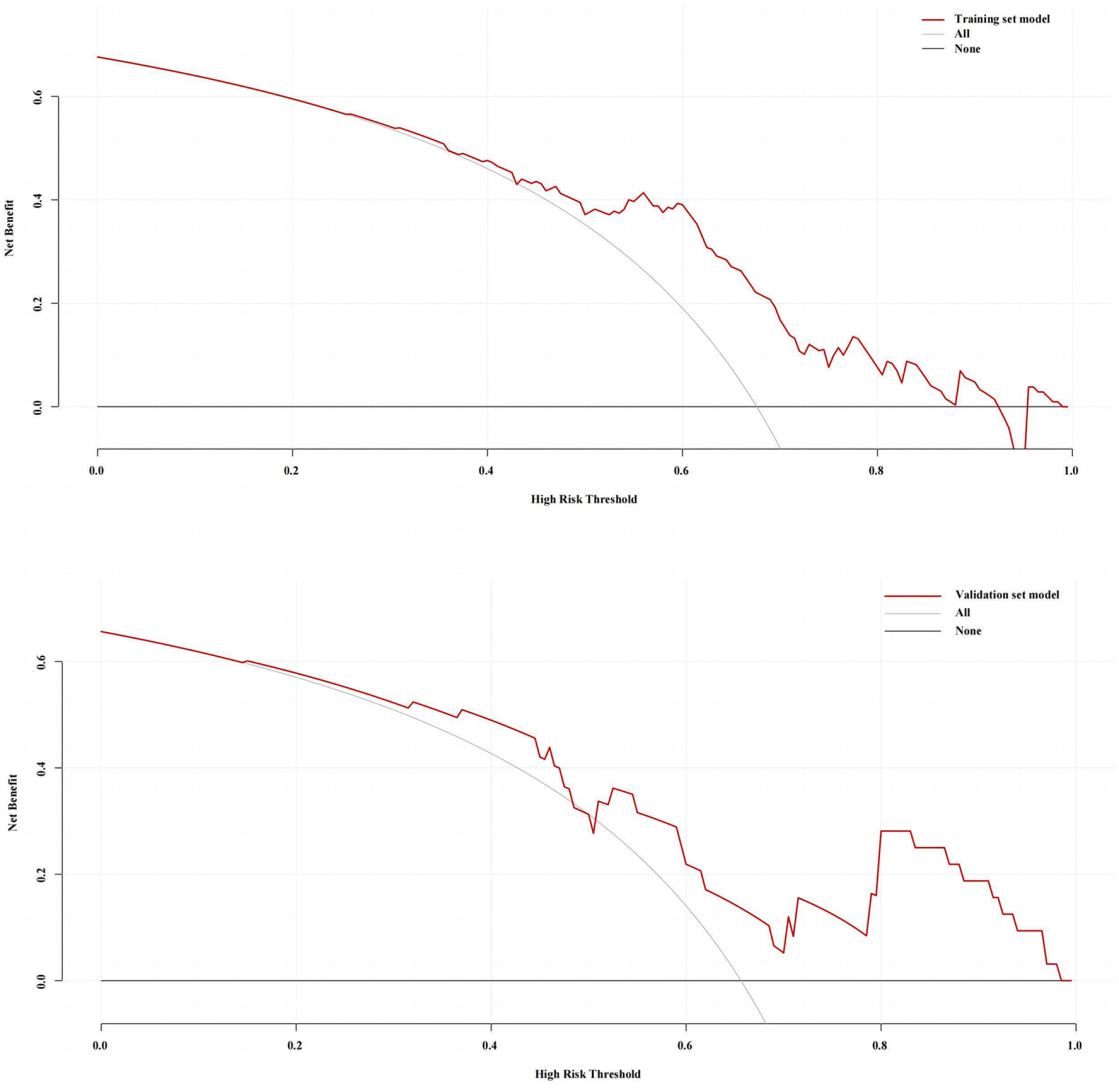
Decision curve analysis (DCA) of the training set and validation set.

The decision curve analysis of the training set and validation set models is shown in Figure 6. The clinical prediction of the model was better when the threshold probabilities of the training set were 24%–93%; and the threshold probabilities of the validation set were 14%–48% and 51%–100%.

## Discussion

Bariatric surgery has become a potent approach to combat obesity. However, the outcomes of weight loss vary greatly among patients with obesity undergoing surgery. Several preoperative variables have been proposed as predictors, but consensus and validation are often lacking (25). Studies have shown that preoperative BMI, sex, and anthropometric indicators can predict the effect of postoperative weight loss (26, 27). Psychological factors, marital relationships, and social factors that influence dietary behavior have also been explored in other studies (28–31). Abdominal adipose tissue is closely related to obesity and obesity related metabolic disorders, but its impact on postoperative weight loss has remained relatively unexplored. Our study aims to determine whether preoperative abdominal fat area and other independent factors can independently predict weight loss after sleeve gastrectomy.

Abdominal fat primarily comprises subcutaneous fat and intra-abdominal fat, among which intra-abdominal fat, also known as visceral fat, is mainly composed of adipose tissue in the peritoneum (such as omentum, mesenteric fat) and retroperitoneal adipose tissue (32). Specific planar imaging images of abdominal fat areas are generally used to represent abdominal fat content in clinical practice. The advantages of single-layer CT or MRI planar measurement of fat area are that it simplifies the measurement process, and minimizes the patient's radiation exposure. The selection of specific planes has also been the focus of research. H Kvist et al. (33) found that the highest correlation between visceral fat area and visceral fat content was found between the fourth and fifth lumbar vertebrae (L4-L5) in CT, and Jennifer L Kuk et al. (34) found that the larger the area of visceral fat obtained between the L2 and L3 suggests that the patient has a higher chance of metabolic syndromes. It has been demonstrated that the fat area in transumbilical plane and the third lumbar vertebral plane (L3) are also strongly associated with abodominal fat content (35–37). Therefore, the third lumbar vertebrae plane (L3) was chosen for analysis, assessing the total abdominal fat area, subcutaneous fat area, visceral fat area and the visceral-to-subcutaneous fat ratio.

As the prevalent index used today, BMI is useful in identifying patients who may have metabolic syndrome and assessing the potential effects of weight loss. But the problem is that it doesn't evaluate whole body fat distribution, which appears to play a pivotal role in weight loss outcomes (38). Physicians cannot determine the precise percentage of body fat in a patient's body weight or the distribution of body fat within the body using BMI. This is the main emphasis of this work, where we included abdominal fat area to account for BMI's lack of accuracy in quantifying body fat distribution. So we included both BMI and abdominal fat distribution characteristics in this study. Binary logistics regression suggested that only TFTA exhibited a formidable and robust predictive efficacy in relation to both %TWL and %EWL.

Some studies have shown that the predictive efficacy of %EWL is greatly affected by preoperative baseline BMI, and it is inaccurate to be used as a recognized index to evaluate the effect of weight loss. %TWL is the best alternative. Therefore, the prediction model of this study is based on %TWL ≥30%. To our knowledge, this is the first study to investigate the association between abdominal fat area and excellent weight loss (≥30%TWL). According to this result, a new model based on BMI and TAFA was explored, which showed improvement in AUC and specificity and sensitivity both in training set and validation set (Figure 4). This study not only verified the model in statistics, but also evaluated the prediction effect from the perspective of clinical benefit. The DCA decision analysis curve was used to conduct a comprehensive analysis of the training group and the verification group, which met the actual requirements of clinical decision making.

Within our cohort, we found that TAFA, VFA, V/S ratio, and SOD were independent predictors of %EWL at 1 year postoperatively (Table 2). It is worth noting that the visceral to subcutaneous fat ratio (V/S ratio) is a hot topic in current studies. It has been shown that an increased visceral to subcutaneous fat ratio is associated with low survival and poor prognosis of various tumors, insulin resistance in type 2 diabetes mellitus population, and incidence of diseases such as gastroesophageal reflux (39–42). In this study, we demonstrated that the area ratio of visceral to subcutaneous fat emerged as an independent predictor of weight loss 1 year after surgery (*p* = 0.011) (Table 2). This underscores the significance of fat distribution in predicting long-term outcomes.

Another novel aspect of our study is that higher preoperative SOD levels predicted improved %EWL. Studies have shown that as adipose tissue accumulates, the activity of antioxidant enzymes, including superoxide dismutase(SOD), catalase(CAT), and glutathione peroxidase(GPx) tends to decrease significantly (43). Chomańska B et al. (44) demonstrated a significant decrease in superoxide dismutase in obese patients as compared to lean controls. After undergoing bariatric surgery, superoxide dismutase (SOD) levels were higher than before (45). Our findings are consistent with this, as we observed a positive correlation between preoperative SOD levels and %EWL 1 year post-surgery, suggesting a potential role for antioxidants in obesity remission. However, further research is imperative to elucidate the specific mechanisms underlying this antioxidant effect.

This research also has some limitations. The present study is a single-center study and has a relatively small sample content. The homogeneity of the study population, comprising solely Chinese individuals, may restrict the generalizability of the findings to other ethnic groups. There were more predictors of excellent weight loss, so more factors need to be included to derive a better predictive model.

This study is significant because it highlights the need to take into account the distribution of adipose tissue in addition to the preoperative baseline weight and BMI when analyzing the effects of weight loss. Preoperative medication and lifestyle intervention of BMI and fat reduction can not only reduce the risk of thromboembolism and reduce systemic inflammatory response during peri-operation period, but also increase the benefit of postoperative weight loss, shorten the hospitalization time and reduce the risk of death (46–48). This is in line with the study's findings, which show that the reduction of BMI and abdominal fat area has positive significance for the maintenance of postoperative weight loss.

The implications of this research are profound, as it underscores the importance of not only considering total body mass but also the distribution of adipose tissue. Tailoring preoperative assessments to include abdominal fat area can assist healthcare professionals in identifying patients with obesity likely to achieve excellent weight loss outcomes. As obesity and its associated metabolic disorders continue to pose a major public health challenge, the insights gained from this study may contribute to the refinement of patient selection and preoperative counseling for those seeking bariatric surgery.

## Conclusion

In this study, we identified abdominal fat area as an independent predictor of excellent weight loss at 1 year postoperatively. We have further introduced a novel model that exhibits superior predictive accuracy based on both BMI and TAFA. While further validation and broader clinical applicability of this index are warranted, these findings underscore the importance of preoperative assessment of abdominal fat area in optimizing postoperative outcomes.

In conclusion, this study has illuminated the significance of abdominal fat area, particularly total abdominal fat area (TAFA), as a robust predictor of substantial weight loss following laparoscopic sleeve gastrectomy (LSG). The development of the novel model, which incorporates TAFA and BMI, has enhanced predictive accuracy and offers promise for optimizing patient selection and counseling in the context of bariatric surgery.

## Data Availability

The raw data supporting the conclusions of this article will be made available by the authors, without undue reservation.

## References

[1] The Lancet Gastroenterology Hepatology. Obesity: another ongoing pandemic. Lancet Gastroenterol Hepatol. (2021) 6(6):411. 10.1016/S2468-1253(21)00143-6 34015350 PMC9259282

[2] Westbury S Oyebode O van Rens T Barber TM. Obesity Stigma: Causes, Consequences, and Potential Solutions. Curr Obes Rep. (2023) 12(1):10–23. 10.1007/s13679-023-00495-3 36781624 PMC9985585

[3] Mingrone G Panunzi S De Gaetano A Guidone C Iaconelli A Nanni G Bariatric-metabolic surgery versus conventional medical treatment in obese patients with type 2 diabetes: 5 year follow-up of an open-label, single-centre, randomised controlled trial. Lancet. (2015) 386(9997):964–73. 10.1016/S0140-6736(15)00075-6 26369473

[4] Angrisani L Santonicola A Iovino P Formisano G Buchwald H Scopinaro N. Bariatric Surgery Worldwide 2013. Obes Surg. (2015) 25(10):1822–32. 10.1007/s11695-015-1657-z 25835983

[5] Cheng Y Huang X Wu D Liu Q Zhong M Liu T Sleeve Gastrectomy with Bypass of Proximal Small Intestine Provides Better Diabetes Control than Sleeve Gastrectomy Alone Under Postoperative High-Fat Diet. Obes Surg. (2019) 29(1):84–92. 10.1007/s11695-018-3520-5 30251097

[6] Di Lorenzo N Antoniou SA Batterham RL Busetto L Godoroja D Iossa A Clinical practice guidelines of the European Association for Endoscopic Surgery (EAES) on bariatric surgery: update 2020 endorsed by IFSO-EC, EASO and ESPCOP. Surg Endosc. (2020) 34(6):2332–58. 10.1007/s00464-020-07555-y 32328827 PMC7214495

[7] Wei M Shao Y Liu QR Wu QZ Zhang X Zhong MW Bile acid profiles within the enterohepatic circulation in a diabetic rat model after bariatric surgeries. Am J Physiol Gastrointest Liver Physiol. (2018) 314(5):G537–G546. 10.1152/ajpgi.00311.2017 29351394

[8] Jiang K Luan H Pu X Wang M Yin J Gong R. Association Between Visceral Adiposity Index and Insulin Resistance: A Cross-Sectional Study Based on US Adults. Front Endocrinol (Lausanne). (2022) 13:921067. 10.3389/fendo.2022.921067 35937809 PMC9353944

[9] de Oliveira Correia ET Mechanick JI Dos Santos Barbetta LM Jorge AJL Mesquita ET. Cardiometabolic-based chronic disease: adiposity and dysglycemia drivers of heart failure. Heart Fail Rev. (2023) 28(1):47–61. 10.1007/s10741-022-10233-x 35368233

[10] Kindel TL Ganga RR Baker JW Noria SF Jones DB Omotosho P Gould JC; ASMBS Quality Improvement and Patient Safety Committee. American Society for Metabolic and Bariatric Surgery: Preoperative Care Pathway for Laparoscopic Roux-en-Y Gastric Bypass. Surg Obes Relat Dis. (2021) 17(9):1529–40. 10.1016/j.soard.2021.05.011 34148848

[11] Robert M Espalieu P Pelascini E Caiazzo R Sterkers A Khamphommala L Efficacy and safety of one anastomosis gastric bypass versus Roux-en-Y gastric bypass for obesity (YOMEGA): a multicentre, randomised, open-label, non-inferiority trial. Lancet. (2019) 393(10178):1299–309. 10.1016/S0140-6736(19)30475-1 30851879

[12] Du Y Zhang J Chen G Sun Z. Formulation and interpretation of the Chinese Guidelines for Surgical Treatment of Obesity and Type 2 Diabetes Mellitus. Biosci Trends. (2021) 15(5):299–304. 10.5582/bst.2021.01287 34334581

[13] Carandina S Soprani A Zulian V Cady J. Long-Term Results of One Anastomosis Gastric Bypass: a Single Center Experience with a Minimum Follow-Up of 10 Years. Obes Surg. (2021) 31(8):3468–75. 10.1007/s11695-021-05455-1 34097238

[14] Grover BT Morell MC Kothari SN Borgert AJ Kallies KJ Baker MT. Defining Weight Loss After Bariatric Surgery: a Call for Standardization. Obes Surg. (2019) 29(11):3493–99. 10.1007/s11695-019-04022-z 31256357

[15] Lehmann A Bobowicz M Lech P Orłowski M Siczewski W Pawlak M Comparison of percentage excess weight loss after laparoscopic sleeve gastrectomy and laparoscopic adjustable gastric banding. Wideochir Inne Tech Maloinwazyjne. (2014) 9(3):351–6. 10.5114/wiitm.2014.44257 25337157 PMC4198654

[16] Turchi MJ Kingma F Laborda N Montanelli A Maldonado JM Fiolo FE. Roux-en-Y gastric bypass in the elderly: is age a determining factor in our outcomes? Surg Obes Relat Dis. (2020) 16(10):1514–20. 10.1016/j.soard.2020.05.015 32665112

[17] Doyle SL Donohoe CL Lysaght J Reynolds JV. Visceral obesity, metabolic syndrome, insulin resistance and cancer. Proc Nutr Soc. (2012) 71(1):181–9. 10.1017/S002966511100320X 22051112

[18] Malietzis G Currie AC Athanasiou T Johns N Anyamene N Glynne-Jones R Influence of body composition profile on outcomes following colorectal cancer surgery. Br J Surg. (2016) 103(5):572–80. 10.1002/bjs.10075 26994716

[19] Irlbeck T Massaro JM Bamberg F O'Donnell CJ Hoffmann U Fox CS. Association between single-slice measurements of visceral and abdominal subcutaneous adipose tissue with volumetric measurements: the Framingham Heart Study. Int J Obes (Lond). (2010) 34(4):781–7. 10.1038/ijo.2009.279 20065971 PMC2982778

[20] Gerken ALH Rohr-Kräutle KK Weiss C Seyfried S Reissfelder C Vassilev G Handgrip Strength and Phase Angle Predict Outcome After Bariatric Surgery. Obes Surg. (2021) 31(1):200–6. 10.1007/s11695-020-04869-7 32803706 PMC7808965

[21] Lutfi R Torquati A Sekhar N Richards WO. Predictors of success after laparoscopic gastric bypass: a multivariate analysis of socioeconomic factors. Surg Endosc. (2006) 20(6):864–7. 10.1007/s00464-005-0115-8 16738971

[22] Manning S Pucci A Carter NC Elkalaawy M Querci G Magno S Early postoperative weight loss predicts maximal weight loss after sleeve gastrectomy and Roux-en-Y gastric bypass. Surg Endosc. (2015) 29(6):1484–91. 10.1007/s00464-014-3829-7 25239175 PMC4422859

[23] Livhits M Mercado C Yermilov I Parikh JA Dutson E Mehran A Is social support associated with greater weight loss after bariatric surgery?: a systematic review. Obes Rev. (2011) 12(2):142–8. 10.1111/j.1467-789X.2010.00720.x 20158617

[24] Clark SM Saules KK Schuh LM Stote J Creel DB. Associations between relationship stability, relationship quality, and weight loss outcomes among bariatric surgery patients. Eat Behav. (2014) 15(4):670–2. 10.1016/j.eatbeh.2014.09.003 25308799

[25] Bylund A Benzein E Sandgren A. Stabilizing family life after gastric bypass surgery. Int J Qual Stud Health Well-being. (2017) 12(1):1325674. 10.1080/17482631.2017.1325674 28741443 PMC5614129

[26] Mårin P Andersson B Ottosson M Olbe L Chowdhury B Kvist H The morphology and metabolism of intraabdominal adipose tissue in men. Metabolism. (1992) 41(11):1242–8. 10.1016/0026-0495(92)90016-4 1435298

[27] Kvist H Chowdhury B Grangård U Tylén U Sjöström L. Total and visceral adipose-tissue volumes derived from measurements with computed tomography in adult men and women: predictive equations. Am J Clin Nutr. (1988) 48(6):1351–61. 10.1093/ajcn/48.6.1351 3202084

[28] Kvist H Chowdhury B Grangård U Tylén U Sjöström L. Total and visceral adipose-tissue volumes derived from measurements with computed tomography in adult men and women: predictive equations. Am J Clin Nutr. (1988) 48(6):1351–61. 10.1093/ajcn/48.6.1351 3202084

[29] Yu AH Duan-Mu YY Zhang Y Wang L Guo Z Yu YQ Correlation between Non-Alcoholic Fatty Liver Disease and Visceral Adipose Tissue in Non-Obese Chinese Adults: A CT Evaluation. Korean J Radiol. (2018) 19(5):923–29. 10.3348/kjr.2018.19.5.923 30174482 PMC6082759

[30] Pedrazzani C Conti C Zamboni GA Chincarini M Turri G Valdegamberi A Impact of visceral obesity and sarcobesity on surgical outcomes and recovery after laparoscopic resection for colorectal cancer. Clin Nutr. (2020) 39(12):3763–70. 10.1016/j.clnu.2020.04.004 32336524

[31] Wirtz TH Loosen SH Schulze-Hagen M Weiskirchen R Buendgens L Abu Jhaisha S CT-based determination of excessive visceral adipose tissue is associated with an impaired survival in critically ill patients. PLoS One. (2021) 16(4):e0250321. 10.1371/journal.pone.0250321 33861804 PMC8051769

[32] Bray GA. Beyond BMI. Nutrients. (2023) 15(10):2254. 10.3390/nu15102254 37242136 PMC10223432

[33] Anyene I Caan B Williams GR Popuri K Lenchik L Giri S Body composition from single versus multi-slice abdominal computed tomography: Concordance and associations with colorectal cancer survival. J Cachexia Sarcopenia Muscle. (2022) 13(6):2974–84. 10.1002/jcsm.13080 36052755 PMC9745558

[34] Feng Z Pang K Tian M Gu X Lin H Yang X Sarcobesity, but not visceral fat, is an independent risk factor for complications after radical resection of colorectal cancer. Front Nutr. (2023) 10:1126127. 10.3389/fnut.2023.1126127 37260520 PMC10228740

[35] Kim EH Kim HK Lee MJ Bae SJ Choe J Jung CH Sex Differences of Visceral Fat Area and Visceral-to-Subcutaneous Fat Ratio for the Risk of Incident Type 2 Diabetes Mellitus. Diabetes Metab J. (2022) 46(3):486–98. 10.4093/dmj.2021.0095 34911174 PMC9171158

[36] Taki Y Sato S Nakatani E Higashizono K Nagai E Nishida M Preoperative skeletal muscle index and visceral-to-subcutaneous fat area ratio are associated with long-term outcomes of elderly gastric cancer patients after gastrectomy. Langenbecks Arch Surg. (2021) 406(2):463–71. 10.1007/s00423-021-02092-1 33515316

[37] Fernández-Sánchez A Madrigal-Santillán E Bautista M Esquivel-Soto J Morales-González A Esquivel-Chirino C Inflammation, oxidative stress, and obesity. Int J Mol Sci. (2011) 12(5):3117–32. 10.3390/ijms12053117 21686173 PMC3116179

[38] Choromańska B Myśliwiec P Dadan J Maleckas A Zalewska A Maciejczyk M. Effects of age and gender on the redox homeostasis of morbidly obese people. Free Radic Biol Med. (2021) 175:108–20. 10.1016/j.freeradbiomed.2021.08.009 34390781

[39] Choromańska B Myśliwiec P Łuba M Wojskowicz P Dadan J Myśliwiec H A Longitudinal Study of the Antioxidant Barrier and Oxidative Stress in Morbidly Obese Patients after Bariatric Surgery. Does the Metabolic Syndrome Affect the Redox Homeostasis of Obese People? J Clin Med. (2020) 9(4):976. 10.3390/jcm9040976 32244612 PMC7230760

[40] Wilding JPH Batterham RL Calanna S Davies M Van Gaal LF Lingvay I Kushner RF; STEP 1 Study Group. Once-Weekly Semaglutide in Adults with Overweight or Obesity. N Engl J Med. (2021) 384(11):989–1002. 10.1056/NEJMoa2032183 33567185

[41] Sun Y Liu B Smith JK Correia MLG Jones DL Zhu Z Association of Preoperative Body Weight and Weight Loss With Risk of Death After Bariatric Surgery. JAMA Netw Open. (2020) 3(5):e204803. 10.1001/jamanetworkopen.2020.4803 32407504 PMC7225906

[42] Mocanu V Marcil G Dang JT Birch DW Switzer NJ Karmali S. Preoperative weight loss is linked to improved mortality and leaks following elective bariatric surgery: an analysis of 548,597 patients from 2015-2018. Surg Obes Relat Dis. (2021) 17(11):1846–53. 10.1016/j.soard.2021.06.021 34330621

